# 

**Published:** 1860-10

**Authors:** 


### CEIPTS FOR THE JOURNAL, FOR SEPTEMBER, 1860.

| | |
|---|---|
| ph Fitch, Ill., vol. 3 | $2 00 |
| Hance, Ill., vol. 3 | 2 00 |
| enan. Ill., vols. 2 and 3 | 4 00 |
| ran, Iowa, vol. 3 | 2 00 |
| Low, Iowa, ½ of vol. 3 | 1 00 |
| W Corey, Ill., 2 and 3 | 4 00 |
| Mills, Wis., vols. 1, 2, and ½ of 3 | 5 00 |
| arr, Ill., vol. 3 | 2 00 |
| ea, Ind., vol. 3 | 2 00 |
| Ross, Ill., vol. 3 | 2 00 |
| er Hard, Ill., vol. 3 | 2 00 |
| ry Folke, " ½ " 3 | 1 00 |
| Hill, Ill., vol. 3 | 2 00 |
| F Cutler, Ill., ¼ of vol 3 | 50 |
| Carter, Ill., vol. 3 | 2 00 |
| Moore, Ind., ½ of 3 and ½ of 4 | 2 00 |
| n Crim, N. T., vol. 3 | 2 00 |
| ove, Iowa, vol. 3 | 2 00 |
| mith, Ill., vol. 3 | 2 00 |
| Blanchard & Castleman, Wis., vol. 3 | 2 00 |
| Adams Co., Med. Society, Miss., vol. 3 | 2 00 |
| C H Quinlan, Ill., vols. 2, 3, and ½ of 4 | 5 00 |
| J Williamson, Iowa, vol. 3 | 2 00 |
| C D Watson, Ind., vols. 2, 3, and ½ of 4 | 5 00 |
| Donaldson & Utley, Ill., vols. 2 and 3 | 4 00 |
| D B Montgomery, Ind., vol. 3 | 2 00 |
| A Eldridge, Ill, vol. 3 | 2 00 |
| Ross W Pierce, Ind., vol. 3 | 2 00 |
| Henry Knapp, Mich., vol. 3 | 2 00 |
| A B Ireland, Iowa, vol. 3 | 2 00 |
| J W Trabue, Ill., vol 3 | 2 00 |
| W Townsend, Ind., vol. 3 | 2 00 |
| J S Skinner, Mich., ½ of 2 and ½ of 3 | 2 00 |
| J B Samuel, Ill., vol 3 | 2 00 |
| C C Warner, Wis., vol. 3 | 2 00 |
| W E Peters, Iowa, vol. 3 | 2 00 |
| A D Reynolds, Iowa, ½ of vol. 3 | 1 00 |

